# Optimization of Biomethane Production via Fermentation of Chicken Manure Using Marine Sediment: A Modeling Approach Using Response Surface Methodology

**DOI:** 10.3390/ijerph182211988

**Published:** 2021-11-15

**Authors:** Fatma Abouelenien, Toyokazu Miura, Yutaka Nakashimada, Nooran S. Elleboudy, Mohammad S. Al-Harbi, Esmat F. Ali, Mustafa Shukry

**Affiliations:** 1 Department of Hygiene and Preventive Medicine, Faculty of Veterinary Medicine, Kafer Elshikh University, Kafrelsheikh 33516, Egypt; fabou2ga@yahoo.com; 2 Unit of Biotechnology, Division of Biological and Life Sciences, Graduate School of Integrated Sciences for Life, Hiroshima University, 1-3-1 Kagamiyama, Higashi-Hiroshima 739-8530, Japan; toyokazumiurato@gmail.com (T.M.); nyutaka@hiroshima-u.ac.jp (Y.N.); 3 Department of Microbiology and Immunology, Faculty of Pharmacy, Ain Shams University, Cairo 11566, Egypt; nooran.elleboudy@pharma.asu.edu.eg; 4 Department of Biology, College of Science, Taif University, P.O. Box 11099, Taif 21944, Saudi Arabia; mharbi@tu.edu.sa (M.S.A.-H.); a.esmat@tu.edu.sa (E.F.A.); 5 Department of Physiology, Faculty of Veterinary Medicine, Kafrelsheikh University, Kafrelsheikh 33516, Egypt

**Keywords:** biomethanation, chicken manure, marine sediment, response surface methodology, optimization

## Abstract

In this study, marine sediment (MS) was successfully used as a source of methanogenic bacteria for the anaerobic digestion (AD) of chicken manure (CM). Using MS showed high production in liquid and semi-solid conditions. Even in solid conditions, 169.3 mL/g volatile solids of chicken manure (VS-CM) was produced, despite the accumulation of ammonia (4.2 g NH_3_-N/kg CM). To the best of our knowledge, this is the highest methane production from CM alone, without pretreatment, in solid conditions (20%). Comparing MS to Ozouh sludge (excess activated sewage sludge) (OS), using OS under semi-solid conditions resulted in higher methane production, while using MS resulted in more ammonia tolerance (301 mL/gVS-CM at 8.58 g NH_3_-N/kg). Production optimization was carried out via a response surface methodology (RDM) model involving four independent variables (inoculum ratio, total solid content, NaCl concentration, and incubation time). Optimized methane production (324.36 mL/gVS-CM) was at a CM:MS ratio of 1:2.5 with no NaCl supplementation, 10% total solid content, and an incubation time of 45 days.

## 1. Introduction

Poultry production is one of the fastest growing industries worldwide. Global poultry production exceeds 130 million tons per year, and increases annually [1]. Every 1000 birds produce approximately 1.2 tons of manure. With around 7 batches per year, 20,000 tons of manure are produced every year [2]. Chicken manure comprises nitrogen (70% uric acid and 30% undigested protein) along with considerable amounts of phosphorus and potassium [3]. The main method of chicken manure disposal is its use as a fertilizer. However, the annual amounts produced exceed the amounts required for the fertilizers needed for agricultural land in many countries [3]. Thus, it is imperative to manage CM properly, lest it cause serious environmental problems, including but not limited to foul smell, pest problems, eutrophication of surface water with a huge amount of nutrients [4], contamination of groundwater with pathogens [5] and heavy metals, and increasing global warming [6].

Using microbial communities for the anaerobic digestion (AD) of chicken manure has been approved as an efficient alternative for disposing of CM via biogas production Methane produced by the co-digestion of CM is a feasible and long-term renewable energy source that can be replicated using kinetic and mathematical models [7]. A variety of feedstocks have been utilized to produce biogas, including energy crops, agricultural and food industry waste products, and municipal waste. However, numerous countries have recommended limiting energy crops due to sustainability and agricultural land issues, and instead focusing on the use of waste and residues for biogas production [8]. AD has been widely used to treat several types of manure, including livestock and swine manure; however, its use for chicken manure treatment has been set back by its high ammonia concentration [9]. Many attempts have been made to mitigate the inhibitory effect of ammonia on AD, including ammonia removal [10] and co-digestion of CM with agriculture waste [11]. Inoculum type, activity, and inoculum-to-substrate ratio are very important factors affecting this technology [12].

Marine sediment (MS), which constitutes 70% of the Earth’s surface, is considered a promising microbial source for AD [13,14]. The collective number of microbial cells in MS is estimated to be between 3 and 6 × 10^29^ cells per gram, decreasing as the depth increases. MS consists of bacteria and archaea, with bacteria being more predominant. *Proteobacteria*—both *Alphaproteobacteria* and *Gammaproteobacteria*—prevail together with *Firmicutes* [15]. Endospores from the families *Bacillaceae*, *Lachnospiraceae*, *Clostridiaceae*, and *Ruminococcaceae*, along with their vegetative forms, heavily populate MS [16]. Miura et al [13] assessed MS from different sources as auspicious microbial sources for the AD of *Saccharina japonica*—a brown alga—at seawater salinity. They concluded that MS could be used as a microbial source for methane production from algae under high-salt conditions, without dilution [12].

Chemometrics originated as a branch of science concerned with using mathematics and statistics to interpret the results of chemical experiments, hence the name chemometrics [17]. Recently, Bystrzanowska [18] referred to it as “an interdisciplinary field that uses mathematical and statistical methods to design or select optimal measurement procedures and experiments and to provide maximum chemical information by analyzing chemical data”. Response surface methodology is one of the most commonly used chemometric tools; it is now preferred for optimization studies, as they have many advantages, including cutting down the number of experiments, therefore saving time, effort, and chemicals, and providing mathematical models for evaluating the statistical significance of factors, both independently and interactively [19]. This is especially important when factors interact significantly; in this case, univariate tools will not provide accurate maxima, and the more significant the interactions, the less precise the univariate optimization results will be; hence, the importance of using statistical models that change variables simultaneously [18].

To the best of the authors’ knowledge, no previous studies have used MS as a source of inoculum for the AD of CM to mitigate ammonia inhibition. Accordingly, the objectives of this study were investigating the use of MS as a source of methanogenic bacteria for the AD of CM, comparing production using MS to that of OS, and applying response surface methodology to optimize methane production using MS, in addition to investigating the tolerance of methanogenic bacteria to the accumulation of ammonia.

## 2. Materials and Methods

### 2.1. Materials

Chicken manure (CM) was collected from belts directly under the chicken cages (cage layer system) at Hiroshima University chicken farm, Hiroshima, Japan. Ozouh sludge (OS), collected from a wastewater treatment center (Hiroshima, Japan), was anaerobically incubated at 55 °C for 60 days at room temperature to achieve complete consumption of available substrates. Marine sediment (MS) was sampled from Hiroshima Bay, Japan. MS was concentrated after centrifugation and removal of supernatant [13].

### 2.2. Characterization of CM, MS, and OS

Total organic carbon (TOC) was determined using a TOC analyzer (TOC-5000, Shimadzu). Total solids (TS); volatile solids (VS); total Kjeldahl nitrogen (TKN); and pH were measured using standard methods [20].

The salinity was calculated from the standard curve of conductivity and the concentration of NaCl [21]. pH was measured by using a pH meter (LAQUAtwin B-712; Horiba, Kyoto, Japan). All tests were conducted in triplicate, and average values are presented in Table 1.

### 2.3. Fermentation Mixtures

Fermentation mixtures (FMs) consisted of CM and OS or MS as sources of anaerobic microorganisms. Water or saline was added to adjust TS%. Aliquots (50 mL) of FM were placed in 125 mL anaerobic serum vials. The anaerobic state was induced by heating in a boiling water bath for 30 min, followed by cooling on ice with continuous bubbling of N_2_:CO_2_ (80:20) for 30 min. The medium was dispensed to the container containing MS, with continuous bubbling of CO_2_. The container was sealed with butyl rubber and incubated at 37 °C. Control conditions with only CM, MS, or OS were included in each run. Incubation was carried out at 35 ± 2 °C for 45 days. Different substrate–inoculum ratios were used, as illustrated in Table 2.

### 2.4. Analytical Methods

Volumes of gases and their composition were monitored every day. Gas production was measured periodically by the displacement of saturated aqueous NaCl in a graduated cylinder. The compositions of CH_4_, H_2_, and CO_2_ were determined using a gas chromatograph (GC-8A, Shimadzu, Japan) equipped with a thermal conductivity detector and a glass column (2 m × 3 mm) packed with C 60/80 Unibeads (Shimadzu, Japan) at 140 °C. Argon was used as the carrier gas, at a pressure of 100 kPa.

When gas production stopped, the vials were opened, and samples were taken to measure their ammonia produced, volatile fatty acids (VFAs), pH, and salinity. Fermentation samples (ca. 0.3 g wet weight) were withdrawn into 2 mL plastic tubes and suspended in 1.2 mL of deionized water. The suspensions were centrifuged at 3000 rpm for 10 min at 4 °C, and the clear supernatants were used to measure pH, ammonia, and volatile fatty acids (VFAs). VFAs were measured using a high-performance liquid chromatograph (Shimadzu, Kyoto, Japan) equipped with an Aminex HPX-87H column (300 mm 7.8 mm, Bio-Rad, Tokyo, Japan). The column temperature was maintained at 65 °C. The flow rate of the mobile phase (0.005 M H_2_SO_4_ solution) was 0.8 mL min^−1^. Ammonia was measured using a commercially available ammonia test kit (Wako Ltd., Osaka, Japan) [21]. All samples were collected in triplicate, and the average values of the three measurements are presented

### 2.5. Effect of Inoculum Size and NaCl Supplementation on the Biomethanation of CM Using MS or OS as the Inoculum Source

A total of 5 anaerobic conditions (three replicates each) (Con)—described as Con1, Con2, Con3, Con4, and Con5—were used in this experiment. In Con1, the substrate–inoculum ratio was 1:30; in Con2, 1:20; Con3, 1:10; Con4, 1:5; and Con5, 1:2.5. In all conditions, the total solid content was 10%. Controls containing only CM, MS, or OS were included. As illustrated in Table 2. All 5 conditions described were tested with and without supplementing the medium with 3% NaCl.

### 2.6. Effect of the Total Solid Content of CM on Biomethanation Using MS as an Inoculum Source

The experiments were conducted at 3 different total solid contents (liquid fermentation 5% TS, semi-solid 10% TS, and solid fermentation 20% TS) at a substrate (CM)–inoculum (MS) ratio of 1: 2.5, as illustrated in Table 3.

### 2.7. Optimization of CM Biomethanation Using MS as an Inoculum Source via the Design of Experiments and Statistical Modeling

Response surface modeling (RSM) was used for experimental design as well as statistical analysis. Design-Expert 7 software (Stat-Ease Inc., Godward St NE, MN, USA) was employed to design experiments, and for data analysis.

An RSM model consisting of 273 experiments was designed. The optimization problem was the need to increase methane gas production by methanogenic bacteria, using chicken manure as an inoculum. The objective function was the amount of methane gas produced via biodegradation using the consortia in chicken manure; however, this was constrained by the accumulation of ammonia, which decreased methane production with increasing inoculum size. The tested parameters were all numerical: substrate–inoculum ratio (1:30, 1:20, 1:10, 1:5, or 1:2.5), incubation time (3–45 days), total solid content (5, 10, or 20%), and the concentration of NaCl (0 or 3%), while the response to be optimized was methane production (mL/g VS). In all experiments, incubation was carried out at 35 ± 2 °C.

### 2.8. Studying the Tolerance of Methanogenic Bacteria in MS to Increasing Ammonia Concentration

In these experiments, MS was used as the inoculum source at a substrate–inoculum ratio of substrate-TS/inoculum-TS = 1:30. TS was equal to 10% ((0.161 g TS of CM + 4.839 g wet weight of MS)/50 g of total weight). The solid content was adjusted using a double-strength dilution medium containing gradually increasing concentrations of ammonia (0, 1.5, 3, 4.5, 6, 7.5, 9, 10.5, 12 g/L). The dilution medium had the following composition (g/L): (NH_4_)_2_SO_4_, 1; Na_2_MoO_4_.2H_2_O, 0.12; Fe(NH_4_)_2_SO_4_.6H_2_O, 0.039; CO(NO_3_)_2_.6H_2_O; 0.29; CaCl_2_.2H_2_O, 0.021; MgSO_4_.7H_2_O, 0.25; NaCl, 30, in addition to Minimum Essential Medium (MEM) vitamin solution (10 mL) and trace element solution (10 mL). After preparation, the dilution media were heated for 30 min, cooled on ice water with N2 bubbling for another 30 min [13], and then distributed in anaerobic vials before adding MS and glucose solution. Glucose solution was filter-sterilized and added at a concentration of 6.44 g/L.

## 3. Results

### 3.1. Effect of Inoculum Size and NaCl Supplementation on Biomethanation of CM Using MS or OS as an Inoculum Source

**Figure 1:**
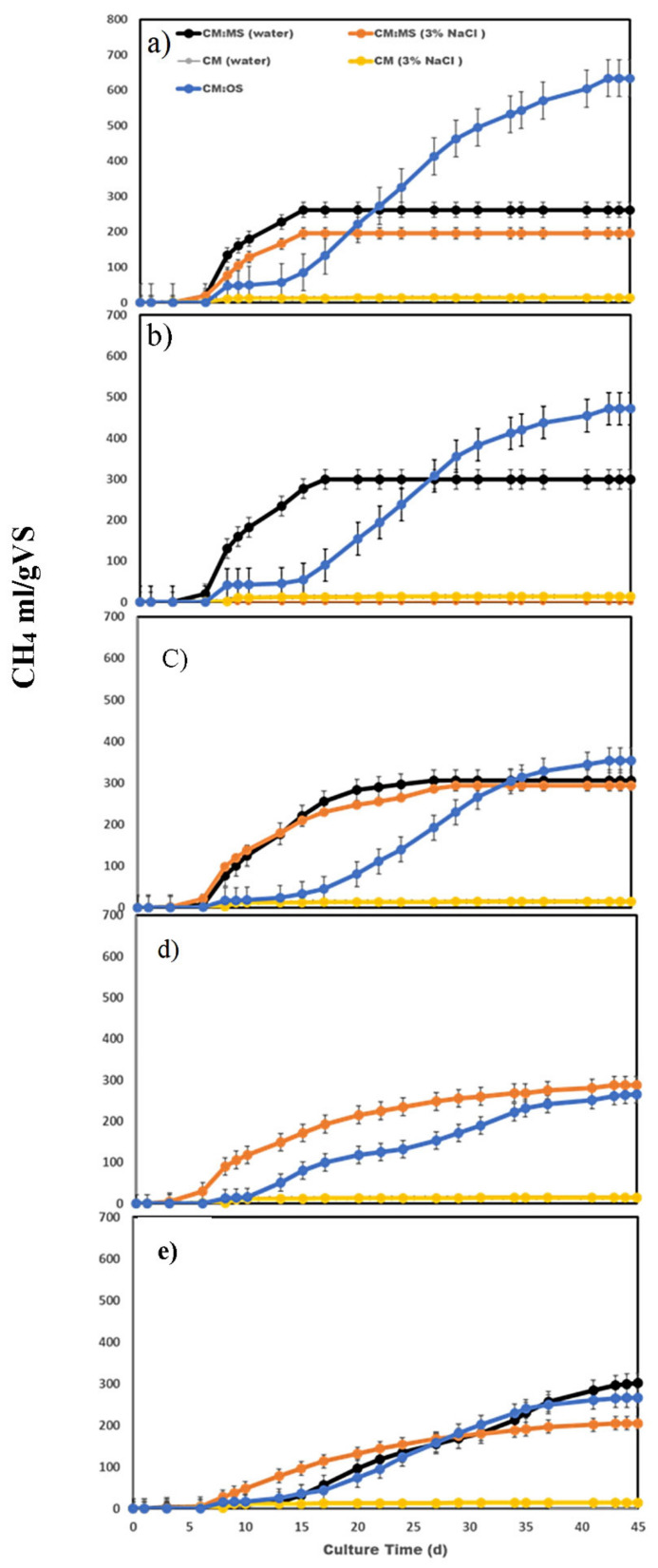
Methane production from anaerobic digestion (10% TS) of CM (chicken manure) using MS (marine sediment) or OS (Ozouh sludge) with different chicken manure–inoculum ratios—CM/MS or OS (TS/TS): (**a**) Condition 1, ratio is 1:30; (**b**) Condition 2, ratio is 1:20; (**c**) Condition 3, ratio is 1:10; (**d**) Condition 4, ratio is 1:5; (**e**) Condition 5, ratio is 1:2.5. Culture time is 45 days; culture temperature = 35 ± 2 °C.

Biogas production at different CM-to-inoculum ratios (OS or MS)—from 1:30 to 1:2.5—as described in conditions 1–5, is shown in Table 4 and Figure 1, the former of which also indicates changes in pH, production of volatile fatty acids (VFAs; acetic and propionic acids), and production of ammonia over the fermentation period. Variable levels of methane production from CM using MS as an inoculum (with or without 3% NaCl supplementation) were recorded, and the results showed successful methane production under mesophilic (35 °C) conditions (Figure 1).

High methane yield (305 mL/g VS-CM) and a final ammonia accumulation as low as 0.78 g NH3-N/kg CM were obtained at CM:MS (1:10). The addition of NaCl had a constructive effect on biomethane production only at the inoculum ratio of 1:10. However, the optimal biomethane production conditions as devised by the RSM models did not include NaCl supplementation.

Comparing methane production from CM under semi-solid conditions using either MS or OS as the inoculum source, it was noted that OS resulted in higher methane production; however, MS use resulted in increased ammonia tolerance, and succeeded in producing methane (301 mL/gVS-CM) despite the accumulation of ammonia (8.58 g NH_3_-N/kg CM)—much higher than the reported inhibitory level. Increasing the inoculum size in the CM–inoculum mixture reduced methane production in the case of OS as the inoculum, while it increased it in the case of MS as the inoculum.

### 3.2. Effect of the Total Solid Content of CM on Biomethanation Using Methanogenic Consortia in MS

**Figure 2:**
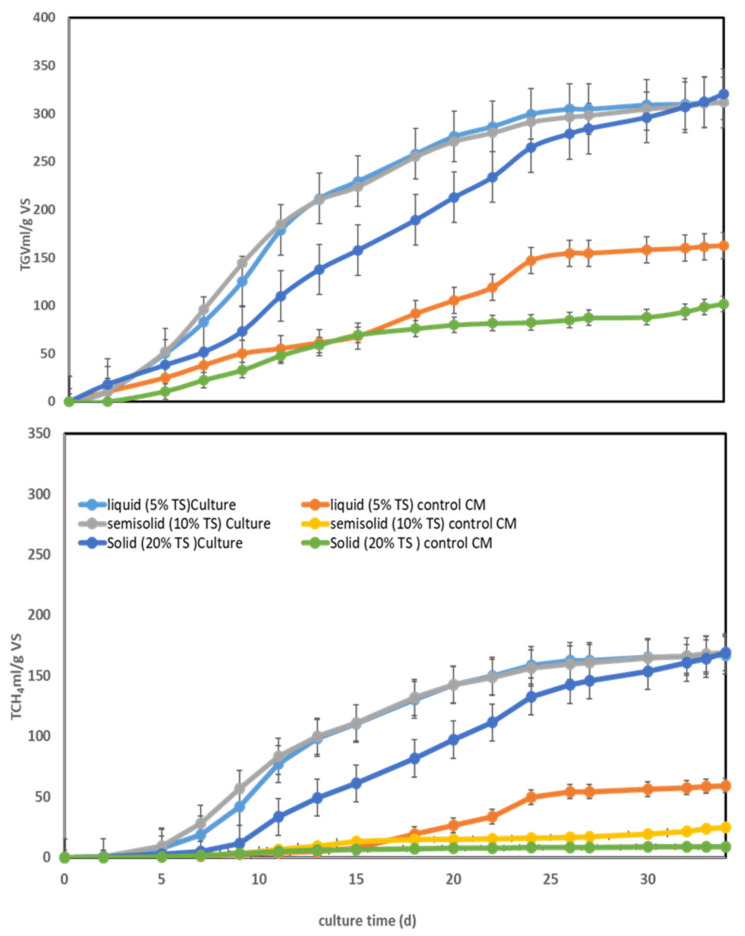
Total biogas volume (TGV) and total methane production (TCH_4_) from anaerobic digestion of CM (chicken manure) using MS (marine sediment) as an inoculum at different TS% values: liquid fermentation (5% TS CM); semi-solid fermentation (10% TS CM); solid fermentation (20% TS CM). Culture time was 34 days, Culture temperature = 35 ± 2 °C. All samples were collected in triplicate, and the average values of the three measurements are presented.

As presented in Table 5 and Figure 2, the increase in biomethane production under both the liquid and semi-solid fermentation conditions started earlier than that in the solid fermentation conditions. However, in all three cases, equivalent maxima were reached at the end of the fermentation process. The changes in pH, VFAs, and NH_3_ content over the fermentation period are also included in Table 5.

In addition, Figure 2 describes the methane production profile at different total solid contents; it can be proposed that MS methanogenic consortia are highly tolerant to the accumulation of ammonia regardless of the total solid percentage. To the best of our knowledge, this is the highest methane production from raw CM under dry conditions (20% TS) without inhibition or VFA accumulation.

### 3.3. Optimization of CM Biomethanation Using MS as an Inoculum Source via the Design of Experiments and Statistical Modeling

The model for the optimization of methane production was a quadratic model using Equation (1). The model experiments with the actual and predicted responses are shown in Table S1 (Supplementary Materials).

Sqrt (Methane production) = 1.29A + 0.50B − 28.38C + 5.67D + 1.49AB + 27.94AC + 0.97AD + 0.81BC + 0.40BD + 4.83A2 − 2.64B2 + 0.21 C2 − 0.94ABD + 1.46 A2B − 6.09A2D + 0.51AB2 − 0.66 B2C − 1.23B2D − 0.97BC2 + 6.33A3 + 2.79B3(1)

where methane production is expressed in mL/g VS, A represents the inoculum ratio, B represents the incubation time (days), C represents the total solid content (%), and D represents the concentration of NaCl (%). Evaluation of the statistical significance of the model was carried out via ANOVA (Table 6). The designed model was highly significant, with model F-values as high as 932.94, and (P model > F) values < 0.0001. This means that there is a less than 0.01% chance that this model would occur due to noise. The model shows a perfect fit, with an R2 value of 0.9908.

**Figure 3:**
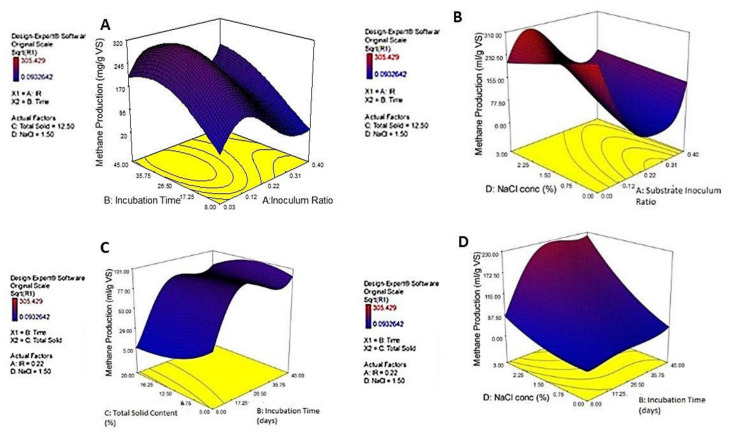
3D response surface plots showing the effects of interaction between every two independent factors when the two other factors are at their midpoint values: (**A**) shows the interaction between (**A**): substrate–inoculum ratio and (**B**): incubation time (days); (**B**) shows the interaction between (**A**): substrate–inoculum ratio and (**D**): NaCl concentration (%); (**C**) shows the interaction between (**B**): incubation time (days) and (**C**): total solid content (%); (**D**) shows the interaction between B: incubation time (days) and (**D**): NaCl concentration (%).

F-values and *p*-values were applied to allocate the significant model terms, as shown in Table 7. The 3D surface and contour plot is shown in Figure 3. The highest *p*-value was recorded for the square term of incubation time, indicating that this variable has the maximal effect on methane production. Chemometric techniques help to specify the optimal values of each variable in order to maximize the studied response. According to this model, the decision variables were optimized methane production (324.36 mL/g VS-CM) at a CM:MS ratio of 1:2.5 without NaCl supplementation, 10% total solid content, and an incubation time of 45 days.

### 3.4. Quality Assurance/Quality Control (QA/QC)

Model validation was performed to confirm that the predicted maxima correlated with the experimental results. Under the optimal conditions, a methane production of 321.73 mL/g VS was obtained—close to the expected value.

### 3.5. Studying the Tolerance of Methanogenic Bacteria in MS to Increasing Concentrations of Ammonia 

Biomethane production, expressed in mL/g glucose utilized by the microorganisms, reduced as ammonia concentration increased—as shown in Table 8, along with the changes in pH, ammonia levels, and VFA levels over the fermentation period. Our findings confirmed no complete inhibition of methane production from glucose by using MS, even at an ammonia concentration of 4.2 g NH_3_-N/kg CM (methane production = 30.5 mL/g glucose). The maximum methane produced when using a glucose medium (130.6 mL/g glucose) was at an ammonia concentration of 0.8 g NH_3_-N/kg CM, with the least amount of final acetate (3.2 mM/g glucose) and a pH of 7.5. pH and acetate accumulation were not profoundly affected by the increase in ammonia concentration (from 1.1 to 4.2 g NH_3_-N/kg CM).

## 4. Discussion

The source of anaerobic microorganisms and the substrate-to-inoculum ratio are critical factors affecting the quality of the AD process [12,22]. Our results revealed the biogas production at different CM–inoculum ratios (OS or MS)—from 1:30 to 1:2.5, as described in conditions 1–5—as shown in Table 4 and Figure 1, the former of which also shows changes in pH and the production of volatile fatty acids (VFAs; acetic and propionic acids) and ammonia over the fermentation period. Variable levels of biomethane production were recorded when culture media containing MS were tested in the presence and absence of 3% NaCl. In addition, using MS as an inoculum resulted in the successful creation of methane from CM as a sole substrate under mesophilic (35 °C) conditions. High methane yield (305 mL/g VS-CM) and final ammonia accumulation as low as 0.78 g NH_3_-N/kg CM were obtained at a CM:MS ratio of 1:10. The addition of NaCl had a constructive effect on biomethane production only at the inoculum size of 1:10. However, the optimal biomethane production conditions as devised by the RSM models did not include NaCl supplementation.

Comparing methane production from CM under semi-solid conditions using either MS or OS as the inoculum source, it was noticed that OS resulted in higher methane production. However, the use of MS resulted in increased ammonia tolerance, and succeeded in producing methane (301 mL/gVS-CM) despite the accumulation of ammonia (8.58 g NH_3_-N/kg CM)—much higher than the reported inhibitory level. While increasing the inoculum size (CM:OS ratio = 1:30) reduced methane production when using OS, it increased methane production when using MS.

In the present study, the use of MS as an inoculum resulted in the successful production of methane from CM as a sole substrate under mesophilic (35 °C) conditions. High methane yield (305 mL/g VS-CM) and final ammonia accumulation as low as 0.78 g NH_3_-N/kg CM were obtained at a CM:MS ratio of 1:10. Marine sediment (MS) was first used by Aspé et al. [23] as a source of anaerobic bacteria to purify fish waste containing high salt content by mixing it with fresh pig manure [23]. Tor et al. [24] stated that the microorganisms living in marine sediments have significant potential for the anaerobic digestion of a variety of organic substrates, and found that sulfate-reducing microorganisms present in MS appear to be involved in the metabolism of both acetate and hydrogen produced by fermentative microorganisms; thus, both fermentative and sulfate-reducing microorganisms cooperate to oxidize glucose.

Miura et al. [13] assessed MS from different sources as auspicious microbial sources for the AD of Saccharina japonica—a brown alga—at seawater salinity; they concluded that MS could be used as a microbial source for the production of methane from algae under high-salt conditions, without dilution [12]. Accordingly, in the present study, NaCl at 3%—the concentration commonly used to simulate seawater salinity [25]—was tested for its effect on the AD process using MS. Our results show that the addition of NaCl had a positive effect on biomethane production only at the inoculum ratio of 1:10. However, the optimal biomethane production conditions as determined by the RSM models did not include NaCl supplementation. This may be because one of the cons of using CM as a substrate is its high concentration of salts (electrical conductivity (EC) = 20 dS/m) and high pH (pH 8.0), which may be expected to inhibit methane production—especially under dry conditions [26,27]. Anwar et al. [28] studied the impact of Na salts on the anaerobic digestion of kitchen waste; they found that as sodium salt concentration increased, the methane yield and the maximal methane production rate decreased, along with the lag-phase time and accumulation of VFAs. Accordingly, they recommended that sodium salt concentration in the anaerobic digesters should be maintained below 8 g/L.

Comparing methane production from CM under semi-solid conditions using either MS or OS as the inoculum source, it was found that OS resulted in higher methane production. Nevertheless, MS use resulted in increased ammonia tolerance, as it succeeded in producing methane (301 mL/GVS CM) despite the accumulation of ammonia (8.58 g NH_3_-N/kg CM)—much higher than the reported inhibitory level [29]. This may be attributed to the buffering action resulting from better degradation of VFAs (acetate), thus preventing the drop in pH (Table 4) [11]. While increasing inoculum size (CM:OS ratio = 1:30) decreased methane production when using OS, it increased methane production when using MS. This result may support the conclusion that MS has superior tolerance to higher ammonia levels compared to OS.

Elasri et al. [30] stated that the energy recovery of CM depends on the waste–inoculum ratio. The maximal biogas production was obtained from a high waste–inoculum ratio (1:7), while the ratio that reached the highest methane percentage was 1:1. Marchioro et al. studied three substrate–inoculum ratios (1:1; 1:1.66, and 1:3) of poultry litter dry AD at 37 °C; they obtained the highest biogas and methane yields of 183 LN biogas.kg−1 VSadd and 74 LN methane.kg−1 VSadd, respectively, obtained at the substrate–inoculum ratio of 1:3 [31]. Abouelenien et al. [32] reported the highest methane yield of 49 mL/g VS when the CM–inoculum ratio was 1:2 at 35 °C, and 103.5 mL/g VS when the CM–inoculum ratio was 1:1 at 55 °C.

As shown in Table 9, the obtained amounts of methane in this study were higher than those reported in previous studies. Cheong et al. [7] produced 396–540 mLg^−1^ VS of methane by co-digesting CM with process effluent from a bioethanol plant (10%; *v*/*v*), and with free ammonia (NH_3_-N; less than 20 mg/L), throughout the experiment. Abouelenien et al. [11] reported the production of 506 CH_4_ mL/g VS from the co-digestion of CM with agricultural waste, with ammonia accumulation of 1.3 g NH_3_-N/kg CM. The solid content of the fermentation medium is one critical factor affecting the overall performance and efficiency of the AD process [5].

It was previously reported that dry AD needs longer lag phases and larger amounts of inoculum than wet digestion systems. Dry AD often results in poor start-up performance due to incomplete mixing and accumulation of VFAs [33,34]. In this study, under dry conditions (20% TS), the highest biogas production (320 mL/g VS-CM) and methane production (169.3 mL/g VS-CM) were much higher than those obtained by the authors of [32], who obtained 31 mL/g VS-CM after an acclimatization period of 254 days. Bujoczek et al. [35] fermented CM at different TS percentages, and they failed to produce methane from CM containing 21.7% TS, even after 120 days of fermentation at 35 °C. Magbanua et al. [36] obtained very low amounts of methane that did not exceed 0.9 mL g^−1^ VS, even after 99 days of batch testing using CM containing TS (17.4%) and VS (14.6%). It can be suggested that MS methanogenic consortia are highly tolerant to the accumulation of ammonia, regardless of the total solid percentage. This is the highest methane production from raw CM under dry conditions (20% TS) without inhibition or VFA accumulation, to the best of our knowledge. This may be attributed to the buffering action generated by the degradation of acetate (0 mM/gVS), which maintains a pH level (8) suitable for AD, despite the ammonia concentration of 4.2 g NH_3_-N/kg CM. Accumulated acetate may have combined with ammonia to form ammonium acetate, resulting in a drop in pH [11,37].

Multivariate statistical tools have recently become the tools of choice for optimization purposes due to their multiple advantages, including reducing the number of performed experiments, thus saving time, effort, and reagents, in addition to providing mathematical models for evaluating the statistical significance of factors, both independently and interactively [19]. This is especially important when factors interact significantly; in this case, univariate tools will not provide accurate maxima, and the more significant the interactions are, the less precise the univariate optimization results will be and, hence, the more important it is to use statistical models that change variables simultaneously [18].

Evaluation of the statistical significance of the model was done through ANOVA (Table 6). The designed model is highly significant, with model F-values as high as 932.94, and (P model > F) values < 0.0001; this means that there is a less than 0.01% chance that the results of this model would occur due to noise. The model shows a perfect fit, with an R2 value of 0.9908, indicating that it can account for 99% of variability [38]; it also possesses a predicted R2 value of 0.9898, indicating the model’s excellent ability to predict the responses of non-experimented values for the test parameters [39]. Adjusted R2 measures the ability of the model to describe variation around the mean, taking into consideration the number of terms included in the model. Accordingly, this value generally decreases as the number of factors that do not add value to the model increases. The model has an adjusted R2 value of 0.9882, indicating that the model would have the ability to explain ~98% of the variability if it were derived from different samples [40].

A model is said to be adequately precise when the value of the parameter “adequate precision” is higher than 4.0. Adequate precision measures signal-to-noise ratio, and reflects the ability of the model to navigate the design space [39]. The model has adequate precision of 48.68, indicating excellent precision. Nevertheless, the ability of a model to produce congruent results when tests are redone under the same conditions is assessed through its “coefficient of variation” (CV), which is calculated by dividing the standard deviation by the mean. The lower the CV value, the more consistent the results are expected to be [41]. The CV values recorded for this model were as low as 10.9%, indicating a reliable model.

F-values and *p*-values were used to assign the significant model terms, as shown in Table 7. The 3D surface and contour plot are shown in Figure 3. The highest *p*-value was recorded for the square term of incubation time, indicating that this variable maximizes methane production; any slight change in its value will be strongly reflected in the amount of methane produced [42].

Under the optimal conditions, a methane production of 321.73 mL/g VS was obtained—close to the predicted value. Li et al. [33] produced ~205 mL/g VS from the semi-solid co-digestion of CM:CS (10.1–11.2% TS) under mesophilic conditions. Additionally, [43] reported the production of 107.25 mL g^−1^ TS (76.92% methane) using a mixture of CM with *Spartina alterniflora* residues (SAR) at 35 °C, with an initial TS of 8%. Miura et al. [21] used acclimatized MS for the biomethanation of raw brown algae, and they obtained methane production of 300 mL/g VS. In all of the aforementioned studies, CM was treated either by co-digestion or by ammonia stripping to enhance methane production, but in the present study CM was used as a sole substrate, without any pretreatment. Additionally, using OS as an inoculum maintains the accumulation of ammonia at all ratios below the inhibitory level of methanogenic bacteria [10,32,33].

Furthermore, Elsayed et al. [44] used three types of inocula—fresh cow manure, activated sludge, and excess sludge—in search of maximal biomethane production from primary sludge (PS) co-digested with fruit and vegetable waste, and recorded that activated sludge was the inoculum source supporting the highest methane yields (141 mL/g VS) [44]. Likewise, Saad et al. [45] tested the effects of using aeration tank sludge, return-activated sludge, and palm oil mill effluent sludge as microbial sources on the anaerobic co-digestion of food waste and CM; they found that the highest biogas production (120.97 N mL/g COD) was obtained when aeration tank sludge was used as the inoculum. In all of the above studies, CM was treated either by co-digestion or ammonia stripping to enhance methane production. Still, CM was used as a sole substrate without any pretreatment in the present study.

Using OS as an inoculum maintains the accumulation of ammonia at all ratios below the inhibitory level of methanogenic bacteria [10,11,32]. This experiment was conducted to investigate the effect of increasing ammonia concentration on biomethane production by methanogenic consortia in MS, using an experimental glucose-containing medium. Biomethane production, expressed in mL/g glucose consumed by the microorganisms, decreased as ammonia concentration increased, as shown in Table 8, along with the changes in pH, ammonia levels, and VFA levels over the fermentation period. Our results confirm the absence of any complete inhibition of methane production from glucose when using MS—even at an ammonia concentration of 4.2 g NH_3_-N/kg CM (methane production = 30.5 mL/g glucose).

Nevertheless, methane production decreased with the increase in ammonia concentration [10]. The highest methane production when using a glucose medium (130.6 mL/g glucose) was at an ammonia concentration of 0.8 g NH_3_-N/kg CM, with the least amount of final acetate (3.2 mM/g glucose) and a pH of 7.5. The most exciting finding in this experiment was that pH and acetate accumulation were not profoundly affected by the increase in ammonia concentration (from 1.1 to 4.2 g NH_3_-N/kg CM). This result supports some buffering action that inhibits the drop in pH and helps to continue methane production.

Practically, the AD of CM is a perfect challenge in the pursuit of renewable sources of energy through biogas production; at the same time, it acts as a way to preserve the environment from contamination. On the other hand, ammonia inhibition is an obstacle to this process, so seeking ammonia-tolerant methanogenic consortia was a practical aim. MS methanogenic consortia, according to this study, seem to be ammonia-tolerant. Additionally, MS is reasonably available, as it constitutes 70% of the Earth’s surface.

Despite the biogas yield from a CM–MS mixture being greater in 20% TS than in any other previous study, the amount of biogas produced under lower TS% (10% and 5% TS) was still lower than that obtained by using Ozouh sludge as the inoculum. Therefore, further improvements in the overall conditions, along with other studies on methanogenic consortia in MS, are needed.

**Table 9:** Summaries of the previous trails for the co-digestion of CM with various organic wastes.

Co- Substrate and/or Inoculum Source	Experimental Conditions						
	RetentionTime (d)	Temperature (°C)	TS%	Ratios of Substrates	Methane (CH_4_) or Biogas Yields	(NH_3_) or (NH_4_+) Values	References
Poultry (P) and hogwastes (H)	- 99	- 35	- 17.4%	- H:P; 0:100	CH_4_ = 0.9 mL g^−1^ VS	1.66 g NH_3_ L^−1^	[36]
Inoculum obtained from sludge(Ozouh) obtained after thermophilic anaerobic digestion of excess activated sludge	254 d254 d	35 °C55 °C	25%25%	- CM–inoculum ratio was 1:2 - CM–inoculum ratio was 1:1	- 49 mLg^−1^ VS methane - 103.5 mLg^−1^ VS methane	- 8 g-N kg−1 CM. - 14 g-N kg−1 CM	[32]
Agriculture wastes (AWS)	Batch I, 2, 3 and 4 = 40 d, 35, 39 and 62 d	35 and 55 °C	10%	CM:AWS is 7:3 (*v*/*v*)	506 CH_4_ mL/gVS	1.3 g NH_3_-N/kg^−1^ CM	[32]
Inoculum obtained from cattle manure under mesophilic conditions	50 d	35 °C	14–32%	(1:1) to (1:7) ratio of CM and inoculum	73.3 NmL g^−1^ VS biogas	-	[7]
Mesophilic acclimated inoculum from a lab-scale CSTR (continuous stirred-tank reactor) with swine and dairy waste water	30 d	37 °C	75%	Poultry litter–inoculum ratios of 1:1; 1:1.66, and 1:3	183 LNbiogas.kg^−1^ VS and 74 L Nmethane.kg^−1^ VS	Below 60 mgNH3.L^−1^	[30]
Process effluent from a bioethanol plant (BPE)	165 d	-	13.2–28.9%	0%, 10%, and 20% (*v*/*v*) of CM:	396–540 mLg^−1^ vs. methane	Less than 20 mgL^−1^	[7]
Marine sediment (MS) or Ozouh sludge(OS) (thermophilic anaerobic digestion of excess activated sludge)	- 45 d	- 35	- 10%	- CM:MS/OS 1:30; 1:20; 1:10; 1:5; 1:2.5, and 1:0	- Using MS resulted in 305 CH_4_ mL/g VS-CM at CM: MS (1:10). using OS resulted in 633.76 CH_4_ mL/g VS-CM at CM: MS (1:30).	- For MS: 0.78 g NH_3_-N/kg^−1^ CM. For OS: 1.02 g NH_3_-N/kg^−1^ CM.	[46]
Marine sediment (MS)	- 34 d	- 35	- 5% 10% - 20%	1:2.5	- Biogas = 320.46 mL/g VS CM - Methane 169.28 mL/g VS CM	4.178 g NH_3_-N/kg^−1^ CM	

## 5. Conclusions

Methanogenic bacteria in MS resulted in successful methane production from CM as a sole substrate, with minimal accumulation of ammonia. Dry CM fermentation using MS resulted in methane production (169.3 mL/g VS-CM), despite the accumulation of ammonia (4.2 g NH_3_-N/kg CM). This is the highest methane production from CM alone, without pretreatment, under solid conditions (20%), to the best of our knowledge. Although using OS resulted in higher methane production under semi-solid conditions, MS resulted in increased ammonia tolerance. Increasing MS inoculum size increased methane production, unlike OS. According to RSM modeling, optimal production (324.36 mL/g VS-CM) was at a CM:MS ratio of 1:2.5, 10% total solid content, and an incubation time of 45 days.

## Figures and Tables

**Table 1:** Characterization of chicken manure (CM), marine sediment (MS), and Ozouh sludge (OS).

Parameter	Unit	Chicken Manure (CM)	Marine Sediment (MS)	Ozouh Sludge (OS)
TS	%W/W	31.9 ± 0.09	52.42 ± 1.8	21.74 ± 0.6
VS	%TS	81.09 ± 1.98	7.385 ± 1.15	52.69 ± 2.61
Water content	%W/W	68.1 ± 0.09	47.58 ± 1.18	78.26 ± 0.6
TOC	g-C kg-TS-1	385 ± 7.06	-	268 ± 7.9
TKN	g-N kg-TS-1	85 ± 2.08	-	32 ± 0.08
Salinity	%	1.68 ± 0.26	1.1 ± 0.11	0.3 ± 0.07

TS: total solids; VS: volatile solids; TOC: total organic carbon; TKN: total Kjeldahl nitrogen.

**Table 2:** Culture conditions for the biomethanation of CM, using MS and OS as inocula.

Condition	Substrate			Inoculum						3% NaCl (mL)	Water (mL)	CM/MS or OS (TS/TS)
	CM (g)	TS (g)	VS (g)	MS (g)	TS (g)	VS (g)	OS (g)	TS (g)	VS (g)			
Con1 ^a^ (−NaCl)	0.505	0.161	0.131	9.231	4.839	0.357					40.26	1/30
Con1 ^b^ (+NaCl)	0.505	0.161	0.131	9.231	4.839	0.357				40.26		1/30
Conc 1	0.505	0.161	0.131				22.26	4.839	2.55		27.24	1/30
Con2 ^a^ (−NaCl)	0.746	0.238	0.193	9.084	4.762	0.352					40.17	1/20
Con2 ^b^ (+NaCl)	0.746	0.238	0.193	9.084	4.762	0.352				40.17		1/20
Conc 2	0.746	0.238	0.193				21.9	4.762	2.509		27.35	1/20
Con3 ^a^ (−NaCl)	1.423	0.455	0.369	8.671	4.545	0.336					39.91	1/10
Con3 ^b^ (+NaCl)	1.423	0.455	0.369	8.671	4.545	0.336				39.91		1/10
Conc 3	1.423	0.455	0.369				20.91	4.545	2.395		27.67	1/10
Con4 ^a^ (−NaCl)	2.609	0.833	0.676	7.949	4.167	0.308					39.44	1/5
Con4 ^b^ (+NaCl)	2.609	0.833	0.676	7.949	4.167	0.308				39.44		1/5
Conc 4	2.609	0.833	0.676				19.17	4.167	2.195		28.22	1/5
Con5 ^a^ (−NaCl)	4.473	1.429	1.158	6.813	3.571	0.264					38.71	1/2.5
Con5 ^b^ (+NaCl)	4.473	1.429	1.158	6.813	3.571	0.264				38.71		1/2.5
Conc 5	4.473	1.429	1.158				16.43	3.571	1.882		29.1	1/2.5
Control ^a^ (−NaCl)	15.66	5	4.055	0	0	0					34.34	1/0
Control ^b^ (+NaCl)	15.66	5	4.055	0	0	0				34.34		1/0

^a^ MS used as inoculum, no saline added; ^b^ MS used as inoculum with 3% NaCl saline was added OS used as inoculum.

**Table 3:** Culture conditions used for the evaluation of methane production at different TS% using MS as an inoculum.

	CM/MS Ratio		CM					MS						Each TS Ratio (%)	
	(TS Basis)	TS (g)	TS (g)	TS of CM (%)	Wet weight (g)	VS%	VS (g)	TS (g)	TS%	Wet Weight (g)	VS%	VS (g)	NaCl (g)	Wet CM	Wet MS
Liquid culture (5%TS)	1/2.5	2.5	0.71	31.94	2.24	25.90	0.58	1.79	52.42	3.41	3.87	0.13	44.36	1.43	3.57
Liquid control (5%TS) (CM)	1/2.5	2.5	0.71	31.94	2.24	25.90	0.58	1.79	52.42	0.94	3.87	0.04	46.83	1.46	-
Liquid control (5%TS) (MS)	1/2.5	2.5	0.71	31.94	2.24	25.90	0.19	1.79	52.42	3.41	3.87	0.13	45.88		3.62
Semi-solid culture (10%TS)	1/2.5	5	1.43	31.94	4.47	25.90	1.16	3.57	52.42	6.81	3.87	0.26	38.71	2.86	7.14
Semi-solid control (10%TS) (CM)	1/2.5	5	1.43	31.94	4.47	25.90	1.16	3.57	52.42	1.87	3.87	0.07	43.65	2.97	
Semi-solid control (10%TS) (MS)	1/2.5	5	1.43	31.94	1.43	25.90	0.37	3.57	52.42	6.81	3.87	0.07	41.76		7.35
Solid culture (20%TS)	1/2.5	10	2.86	31.94	8.95	25.90	2.32	7.14	52.42	13.63	3.87	0.53	27.43	5.71	14.29
Solid control (20%TS) (CM)	1/2.5	10	2.86	31.94	8.95	25.90	2.32	7.14	52.42	3.74	3.87	0.15	37.31	6.18	
Solid control (20%TS) (MS)	1/2.5	10	2.86	31.94	2.86	25.90	0.74	7.14	52.42	13.63	3.87	0.53	33.52		15.15

**Table 4:** Methane production, along with initial and final changes in pH, VFAs (acetate and propionate), and ammonia levels during anaerobic digestion of CM using MS or OS as the inoculum with different CM–inoculum ratios under semi-solid conditions (TS% 10) (±SD).

Conditions	pH		VFAs (mM/g VS)				NH_3_ gN/kg CM		H mL/g VS-CM
			Acetate		Propionate				
	Initial	Final	Initial	Final	Initial	Final	Initial	Final	
Con1 ^a^ (−NaCl)	7.8 ± 0	7.65 ± 0.07	0	0	0	0	0.04 ± 0.02	0.41 ± 0.09	261.88 ± 9.86
Con1 ^b^ (+NaCl)	7.85 ± 0.07	7.4 ± 0	0	0	0	0	0.09 ± 0.04	1.53 ± 0.06	195.67 ± 72.4
Con1 c	6.85 ± 0.07	7.6 ± 0.14	0	0	0	0	0.29 ± 0.14	1.02 ± 1	633.76 ± 18. 69
Con2 ^a^ (−NaCl)	7.85 ± 0.14	7.65 ± 0.07	0	0	0	0	0.07 ± 0	0.40 ± 0.12	299.14 ± 7.09
Con2 ^b^ (+NaCl)	7.7 ± 0	8.15 ± 0.64	0	0	0	0	0.10 ± 0.03	0.70 ± 0.35	0
Con2 ^c^	6.85 ± 0.064	7.6 ± 0	0	0	0	0	0.22 ± 0.14	1.09 ± 0.04	471.41 ± 4.63
Con3 ^a^ (−NaCl)	7.75 ± 0.21	7.65 ± 0.07	0	0	0	0	0.21 ± 0.03	0.78 ± 0.11	305.43 ± 6.86
Con3 ^b^ (+NaCl)	7.85 ± 0.07	7.65 ± 0.07	0	0	0	0	0.21 ± 0.02	0.77 ± 0.07	293.8 ± 2.42
Con3 ^c^	7.25 ± 0.07	7.75 ± 0.07	4 ± 5.65	0	0	0	0.39 ± 0.05	1.22 ± 0.06	354.12 ± 16.65
Con4 ^a^ (−NaCl)	7.3 ± 0.0	7.85 ± 0.07	23.56 ± 1.7	1.85 ± 0.64	3.94 ± 0.31	1.53 ± 0.54	0.56 ± 0.04	1.38 ± 0.04	13.12 ± 5.84
Con4 ^b^ (+NaCl)	7.4 ± 0.0	7.7 ± 0	18.25 ± 4.03	0.545 ± 0.70	3.93 ± 0.4	0	0.54 ± 0.07	1.09 ± 0.12	287.53 ± 3.15
Con4 ^c^	7.15 ± 0.07	7.75 ± 0.07	27.88 ± 4.54	0.35 ± 0.49	2.465 ± 1.46	0	0.88 ± 0.23	1.25 ± 0.38	265.79 ± 31.55
Con5 ^a^ (−NaCl)	6.8 ± 0.07	7.9 ± 0	56.12 ± 8.41	3.81 ± 3.5	5.97 ± 0.27	1.6 ± 2.29	4.87 ± 0.5	8.58 ± 1.28	301.22 ± 1.57
Con5 ^b^ (+NaCl)	7.3 ± 0.14	7.85 ± 0.07	44.06 ± 1.75	2.2 ±1.82	7.49 ± 0.56	0	5.11 ± 1.46	7.95 ± 0.75	205.08 ± 21.18
Con5 ^c^	6.95 ± 0.07	7.85 ± 0.07	62.61 ± 2.39	3.16 ± 3.5	8.48 ± 0.71	0	1.08 ± 0.14	2.36 ± 0.12	266.41 ± 7.9
Control ^a^ (−NaCl)	7.1 ± 0.14	7.2 ± 0.28	230.85 ± 18.45	310.9 ± 28.28	30.4 ± 1.84	22.38 ± 3.4	1.01 ± 0.03	3.18 ± 0.1	14.05 ± 1.16
Control ^b^ (+NaCl)	7.1 ± 0.42	7.25 ± 0.07	208.25 ± 31.32	304.7 ± 37.96	26.59 ± 4.57	27.33 ± 4.57	1.36 ± 0.06	1.90 ± 0.12	13.78 ± 1.92

^a^: MS was used as inoculum, no saline was added; ^b^: MS was used as inoculum and 3% NaCl saline was added; ^c^: OS was used as inoculum. Culture time was 45 days; culture temperature = 35 ± 2 °C.

**Table 5:** Initial and final changes in pH, VFAs (acetate and propionate), and ammonia during the anaerobic digestion of CM using MS as the inoculum, under different TS% (liquid fermentation, 5% TS; semi-solid, 10% TS; and solid fermentation, 20% TS (±SD)).

Culture Condition	pH		VFAs (mM/Kg CM)				NH_3_ gN/kg CM		TGV mL/g VS	CH_4_ mL/g VS-CM
			Acetate		Propoinate					
	Initial	Final	Initial	Final	Initial	Final	Initial	Final		
Liquid (5% TS) culture	7.35 ± 0.07	7.8 ± 0	7.14 ± 0.54	0	0	0	0.694 ± 0	1.530 ± 0.32	311.74 ± 28.09	166.96 ± 17.82
Liquid (5% TS) control (CM)	7.3 ± 0	7.6 ± 0	2.055 ± 1.9	0	0	0	0.62 ± 0.04	0.88 ± 0.12	162.35 ± 53.74	59.25 ± 35.74
Liquid (5% TS) control (MS)	7.2 ± 0	7.5 ± 0	0	0	0	0	0.02 ± 0.01	0.008 ± 0.005	0	0
Semi-solid (10% TS) culture	7.35 ± 0.21	7.85 ± 0.07	17.79 ± 3.2	5.39 ± 4.5	4.115 ± 0.89	0	1.26 ± 0.22	1.7 ± 0.12	312.18 ± 36.03	168.79 ± 18.7
Semi-solid(10% TS) control (CM)	7.45 ± 0.21	7.55 ± 0.21	6.85 ± 4.6	20.98 ± 4.87	1.525 ± 1.15	4.09 ± 3.03	0.89 ± 0.03	2.15 ± 0.48	101.9 ± 1.83	24.89 ± 1.05
Semi-solid(10% TS) control (MS)	7.5 ± 0	7.7 ± 0.14	0	0	0	0	0.01 ± 0.005	0.009 ± 0.001	0	0
Solid (20%TS) culture	7.1 ± 0.14	8 ± 0.14	41.07 ± 6.6	0	9.51 ± 0.21	4.49 ± 3.06	2.12 ± 0.25	4.18 ± 1.38	320.46 ± 26.6	169.28 ± 21.11
Solid (20%TS) control (CM)	7.35 ± 0.07	7.2 ± 0.14	35.04 ± 0.8	80.67 ± 3.35	6.5 ± 0.27	14.9 ± 0.06	2.14 ± 0.04	3.73 ± 0.02	101.9 ± 0.91	8.62 ± 1.1
Solid (20%TS) control (MS)	7.65 ± 0.07	7.75 ± 0.07	0	0	0	0	0.001 ± 0	0.008 ± 0.004	0	0

Culture time = 34 d; culture temperature = 35 ± 2 °C. VS: volatile solids.

**Table 6:** ANOVA for the quadratic model.

Source	Sum of Squares	Degrees of Freedom	Mean Square	F Value	Prob > F	
Model	32,694.60	21	1556.89	932.94	<0.0001	Significant
Residual	302.05	181	1.67			
Lack of fit	229.94	168	0.25	0.25	1.0000	Not significant
Pure error	72.11	13	5.55			
Total	32,996.65	202				
R-squared = 0.9764						
Adjusted R-squared = 0.9741 Predicted R-squared = 0.9731						
Adequate precision = 36.662 CV = 17.76%						

**Table 7:** Coefficient estimates, standard errors, F-values, and p-values of the model terms for optimizing total solids.

Source	Coefficient Estimate	Standard Error	F Value	*p*-Value	
A-IR	1.29	1.09	1.40	0.2385	
B-Time	0.50	0.58	0.75	0.3884	
C-Total Solid	−28.38	0.83	1161.94	<0.0001	Significant
D-NaCl	5.67	0.23	596.43	<0.0001	Significant
AB	1.49	0.19	58.37	<0.0001	Significant
AC	27.94	0.92	926.62	<0.0001	Significant
AD	0.97	0.13	58.37	<0.0001	Significant
BC	0.81	0.93	0.77	0.3816	
BD	0.40	0.15	7.12	0.0083	Significant
A2	4.83	0.26	332.03	<0.0001	Significant
B2	−2.64	0.52	25.78	<0.0001	Significant
C2	0.21	0.48	0.20	0.6561	
ABD	−0.94	0.19	25.47	<0.0001	Significant
A2B	1.46	0.36	16.47	<0.0001	Significant
A2D	−6.09	0.25	612.00	<0.0001	Significant
AB2	0.51	0.32	2.56	0.1114	
B2C	−0.66	1.38	0.23	0.6316	
B2D	−1.23	0.24	25.54	<0.0001	Significant
BC2	−0.97	0.84	1.32	0.2530	
A3	6.33	0.93	45.85	<0.0001	Significant
B3	2.79	0.42	45.07	<0.0001	Significant

A: substrate: inoculum ratio; B: incubation time (days); C: total solid content (%); and D: concentration of NaCl (%).

**Table 8:** Changes in pH, ammonia, and VFAs from the AD of glucose media, using MS as the inoculum, increase ammonia levels (±SD).

NH_4_ (g/L)	pH		NH_3_ Conc. (g/L)		Propionate (mM/L)		Acetate (mM/L)		CH_4_ (mL/g Glucose)
	Initial	Final	Initial	Final	Initial	Final	Initial	Final	
0	8.2 ± 0.07	7.55 ± 0	0.27 ± 0.04	0.27 ± 0.14	0	8 ± 1.28	0	23.48 ± 4.7	80.86 ± 2.73
1.5	8.1 ± 0.07	7.55 ± 0	0.64 ± 0.23	0.80 ± 0.23	0	8.48 ± 1.02	0	3.2 ± 0.5	130.62 ± 13.44
3	8.2 ± 0.02	7.5 ± 0.07	1.14 ± 0.04	1.17 ± 0.47	0	3.89 ± 0.12	0	13.79 ± 1.8	125.76 ± 8.16
4.5	8.15 ± 0.21	7.45 ± 0.07	1.6 ± 0.09	1.4 ± 0.14	0	2.82 ± 0.07	0	30.8 ± 5.6	47.80 ± 17
6	8.2 ± 0.07	7.4 ± 0.12	1.89 ± 0.12	2.07 ± 0.12	0	4.39 ± 0.49	0	26.54 ± 4.5	76.81 ± 1.14
7.5	8.05 ± 0.02	7.45 ± 0.14	2.42 ± 0.12	2.50 ± 0.02	0	4.35 ± 0.33	0	29.66 ± 1.05	70.42 ± 0.49
9	8.1 ± 0.14	7.45 ± 0.01	3.24 ± 0.1	3.05 ± 0.1	0	2.82 ± 0.21	0	34.59 ± 1.82	63.26 ± 5.05
10.5	7.95 ± 0	7.45 ± 0	3.52 ± 0.14	3.58 ± 0.03	0	2.08 ± 0.12	0	35.92 ± 1.07	47.25 ± 18.72
12	7.9 ± 0.12	7.45 ± 0	3.80 ± 0.19	4.18 ± 0.5	0	1.52 ± 0.05	0	30.78 ± 1.55	30.45 ± 11.45

Culture time was 31 days; culture temperature = 35 ± 2 °C.

## Data Availability

Data are available upon request.

## Supplementary Materials

The following are available online at https://www.mdpi.com/article/10.3390/ijerph182211988/s1, Table S1: Experimental design for optimization of inoculum with actual and predicted values of methane production.
