# Supplementary material for: Optimization of Biomethane Production via Fermentation of Chicken Manure Using Marine Sediment: A Modeling Approach Using Response Surface Methodology
Source: Int J Environ Res Public Health. 2021 Nov 15;18(22):11988. doi: 10.3390/ijerph182211988 (PMC8622348; doi:10.3390/ijerph182211988)
Supplement: Supplementary file 1 [file ijerph-18-11988-s001.zip › ijerph-1383962-supplementary.pdf]

Table S1. Experimental design for optimization of inoculum with actual and predicted values of methane production.

| Exp. | Inoculum Ratio | Time (days) | Inoculum | Supplementation with NaCl | Actual Value* | Predicted Value* | Residual |
|---|---|---|---|---|---|---|---|
| **1** | 1: 30 | 8 | MS | N S | 11.61 | 13.98 | -2.37 |
| **2** | 1: 30 | 9 | MS | N S | 12.69 | 14.38 | -1.70 |
| **3** | 1: 30 | 10 | MS | N S | 13.42 | 14.77 | -1.35 |
| **4** | 1: 30 | 13 | MS | N S | 15.10 | 15.79 | -0.70 |
| **5** | 1: 30 | 15 | MS | N S | 16.18 | 16.38 | -0.20 |
| **6** | 1: 30 | 17 | MS | N S | 16.18 | 16.90 | -0.71 |
| **7** | 1: 30 | 20 | MS | N S | 16.18 | 17.54 | -1.35 |
| **8** | 1: 30 | 22 | MS | N S | 16.18 | 17.88 | -1.69 |
| **9** | 1: 30 | 24 | MS | N S | 16.18 | 18.15 | -1.97 |
| **10** | 1: 30 | 27 | MS | N S | 16.18 | 18.44 | -2.26 |
| **11** | 1: 30 | 29 | MS | N S | 16.18 | 18.55 | -2.37 |
| **12** | 1: 30 | 31 | MS | N S | 16.18 | 18.61 | -2.43 |
| **13** | 1: 30 | 34 | MS | N S | 16.18 | 18.59 | -2.41 |
| **14** | 1: 30 | 35 | MS | N S | 16.18 | 18.56 | -2.38 |
| **15** | 1: 30 | 37 | MS | N S | 16.18 | 18.45 | -2.27 |
| **16** | 1: 30 | 41 | MS | N S | 16.18 | 18.09 | -1.91 |
| **17** | 1: 30 | 43 | MS | N S | 16.18 | 17.84 | -1.66 |
| **18** | 1: 30 | 44 | MS | N S | 16.18 | 17.70 | -1.51 |
| **19** | 1: 30 | 45 | MS | N S | 16.18 | 17.54 | -1.36 |
| **20** | 1: 30 | 8 | MS | S | 8.81 | 8.49 | 0.31 |
| **21** | 1: 30 | 9 | MS | S | 10.27 | 8.99 | 1.28 |
| **22** | 1: 30 | 10 | MS | S | 11.35 | 9.46 | 1.89 |
| **23** | 1: 30 | 13 | MS | S | 12.88 | 10.76 | 2.13 |
| **24** | 1: 30 | 15 | MS | S | 13.99 | 11.52 | 2.47 |
| **25** | 1: 30 | 17 | MS | S | 13.99 | 12.19 | 1.80 |
| **26** | 1: 30 | 20 | MS | S | 13.99 | 13.05 | 0.93 |
| **27** | 1: 30 | 22 | MS | S | 13.99 | 13.53 | 0.46 |
| **28** | 1: 30 | 24 | MS | S | 13.99 | 13.94 | 0.05 |
| **29** | 1: 30 | 27 | MS | S | 13.99 | 14.41 | -0.42 |
| **30** | 1: 30 | 29 | MS | S | 13.99 | 14.63 | -0.65 |
| **31** | 1: 30 | 31 | MS | S | 13.99 | 14.79 | -0.80 |
| **32** | 1: 30 | 34 | MS | S | 13.99 | 14.91 | -0.92 |
| **33** | 1: 30 | 35 | MS | S | 13.99 | 14.92 | -0.93 |
| **34** | 1: 30 | 37 | MS | S | 13.99 | 14.89 | -0.90 |
| **35** | 1: 30 | 41 | MS | S | 13.99 | 14.65 | -0.67 |
| **36** | 1: 30 | 43 | MS | S | 13.99 | 14.45 | -0.47 |

| | | | | | | | |
|---|---|---|---|---|---|---|---|
| **37** | 1: 30 | 44 | MS | S | 13.99 | 14.33 | -0.35 |
| **38** | 1: 30 | 45 | MS | S | 13.99 | 14.20 | -0.21 |
| **39** | 1: 30 | 8 | OS | N S | 6.85 | 4.17 | 2.69 |
| **40** | 1: 30 | 9 | OS | N S | 6.96 | 5.06 | 1.91 |
| **41** | 1: 30 | 10 | OS | N S | 7.04 | 5.92 | 1.12 |
| **42** | 1: 30 | 13 | OS | N S | 7.59 | 8.39 | -0.80 |
| **43** | 1: 30 | 15 | OS | N S | 9.24 | 9.93 | -0.69 |
| **44** | 1: 30 | 17 | OS | N S | 11.54 | 11.38 | 0.16 |
| **45** | 1: 30 | 20 | OS | N S | 14.89 | 13.40 | 1.49 |
| **46** | 1: 30 | 22 | OS | N S | 16.51 | 14.64 | 1.87 |
| **47** | 1: 30 | 24 | OS | N S | 18.05 | 15.81 | 2.24 |
| **48** | 1: 30 | 27 | OS | N S | 20.32 | 17.43 | 2.89 |
| **49** | 1: 30 | 29 | OS | N S | 21.52 | 18.42 | 3.11 |
| **50** | 1: 30 | 31 | OS | N S | 22.23 | 19.33 | 2.90 |
| **51** | 1: 30 | 34 | OS | N S | 23.07 | 20.58 | 2.49 |
| **52** | 1: 30 | 35 | OS | N S | 23.31 | 20.96 | 2.34 |
| **53** | 1: 30 | 37 | OS | N S | 23.88 | 21.68 | 2.19 |
| **54** | 1: 30 | 41 | OS | N S | 24.58 | 22.94 | 1.64 |
| **55** | 1: 30 | 43 | OS | N S | 25.17 | 23.49 | 1.69 |
| **56** | 1: 30 | 44 | OS | N S | 25.17 | 23.74 | 1.44 |
| **57** | 1: 30 | 45 | OS | N S | 25.17 | 23.97 | 1.20 |
| **58** | 1: 20 | 8 | MS | N S | 11.41 | 12.91 | -1.50 |
| **59** | 1: 20 | 9 | MS | N S | 12.67 | 13.31 | -0.64 |
| **60** | 1: 20 | 10 | MS | N S | 13.50 | 13.68 | -0.18 |
| **61** | 1: 20 | 13 | MS | N S | 15.29 | 14.69 | 0.60 |
| **62** | 1: 20 | 15 | MS | N S | 16.65 | 15.27 | 1.38 |
| **63** | 1: 20 | 17 | MS | N S | 17.30 | 15.78 | 1.52 |
| **64** | 1: 20 | 20 | MS | N S | 17.30 | 16.40 | 0.89 |
| **65** | 1: 20 | 22 | MS | N S | 17.30 | 16.74 | 0.56 |
| **66** | 1: 20 | 24 | MS | N S | 17.30 | 17.00 | 0.29 |
| **67** | 1: 20 | 27 | MS | N S | 17.30 | 17.29 | 0.01 |
| **68** | 1: 20 | 29 | MS | N S | 17.30 | 17.40 | -0.10 |
| **69** | 1: 20 | 31 | MS | N S | 17.30 | 17.45 | -0.16 |
| **70** | 1: 20 | 34 | MS | N S | 17.30 | 17.43 | -0.13 |
| **71** | 1: 20 | 35 | MS | N S | 17.30 | 17.40 | -0.10 |
| **72** | 1: 20 | 37 | MS | N S | 17.30 | 17.29 | 0.01 |
| **73** | 1: 20 | 41 | MS | N S | 17.30 | 16.93 | 0.37 |
| **74** | 1: 20 | 43 | MS | N S | 17.30 | 16.68 | 0.62 |
| **75** | 1: 20 | 44 | MS | N S | 17.30 | 16.54 | 0.76 |
| **76** | 1: 20 | 45 | MS | N S | 17.30 | 16.38 | 0.91 |
| **77** | 1: 20 | 8 | OS | N S | 6.47 | 4.69 | 1.79 |

| | | | | | | | |
|---|---|---|---|---|---|---|---|
| **78** | 1: 20 | 9 | OS | N S | 6.50 | 5.55 | 0.95 |
| **79** | 1: 20 | 10 | OS | N S | 6.53 | 6.38 | 0.15 |
| **80** | 1: 20 | 13 | OS | N S | 6.71 | 8.76 | -2.05 |
| **81** | 1: 20 | 15 | OS | N S | 7.39 | 10.24 | -2.85 |
| **82** | 1: 20 | 17 | OS | N S | 9.49 | 11.63 | -2.14 |
| **83** | 1: 20 | 20 | OS | N S | 12.43 | 13.57 | -1.13 |
| **84** | 1: 20 | 22 | OS | N S | 13.94 | 14.76 | -0.82 |
| **85** | 1: 20 | 24 | OS | N S | 15.44 | 15.87 | -0.43 |
| **86** | 1: 20 | 27 | OS | N S | 17.56 | 17.41 | 0.15 |
| **87** | 1: 20 | 29 | OS | N S | 18.86 | 18.34 | 0.51 |
| **88** | 1: 20 | 31 | OS | N S | 19.59 | 19.21 | 0.38 |
| **89** | 1: 20 | 34 | OS | N S | 20.29 | 20.38 | -0.09 |
| **90** | 1: 20 | 35 | OS | N S | 20.48 | 20.74 | -0.25 |
| **91** | 1: 20 | 37 | OS | N S | 20.93 | 21.41 | -0.48 |
| **92** | 1: 20 | 41 | OS | N S | 21.32 | 22.57 | -1.25 |
| **93** | 1: 20 | 43 | OS | N S | 21.71 | 23.07 | -1.36 |
| **94** | 1: 20 | 44 | OS | N S | 21.71 | 23.30 | -1.58 |
| **95** | 1: 20 | 45 | OS | N S | 21.71 | 23.51 | -1.80 |
| **96** | 1: 10 | 8 | MS | N S | 8.72 | 9.43 | -0.71 |
| **97** | 1: 10 | 9 | MS | N S | 10.04 | 9.82 | 0.22 |
| **98** | 1: 10 | 10 | MS | N S | 11.16 | 10.19 | 0.98 |
| **99** | 1: 10 | 13 | MS | N S | 13.31 | 11.19 | 2.12 |
| **100** | 1: 10 | 15 | MS | N S | 14.85 | 11.76 | 3.09 |
| **101** | 1: 10 | 17 | MS | N S | 15.98 | 12.26 | 3.72 |
| **102** | 1: 10 | 20 | MS | N S | 16.84 | 12.88 | 3.95 |
| **103** | 1: 10 | 22 | MS | N S | 17.04 | 13.22 | 3.82 |
| **104** | 1: 10 | 24 | MS | N S | 17.23 | 13.49 | 3.75 |
| **105** | 1: 10 | 27 | MS | N S | 17.48 | 13.78 | 3.70 |
| **106** | 1: 10 | 29 | MS | N S | 17.48 | 13.90 | 3.58 |
| **107** | 1: 10 | 31 | MS | N S | 17.48 | 13.96 | 3.52 |
| **108** | 1: 10 | 34 | MS | N S | 17.48 | 13.95 | 3.52 |
| **109** | 1: 10 | 35 | MS | N S | 17.48 | 13.93 | 3.55 |
| **110** | 1: 10 | 37 | MS | N S | 17.48 | 13.83 | 3.64 |
| **111** | 1: 10 | 41 | MS | N S | 17.48 | 13.51 | 3.97 |
| **112** | 1: 10 | 43 | MS | N S | 17.48 | 13.28 | 4.19 |
| **113** | 1: 10 | 44 | MS | N S | 17.48 | 13.15 | 4.32 |
| **114** | 1: 10 | 45 | MS | N S | 17.48 | 13.01 | 4.46 |
| **115** | 1: 10 | 8 | MS | S | 9.90 | 12.17 | -2.27 |
| **116** | 1: 10 | 9 | MS | S | 10.89 | 12.62 | -1.73 |
| **117** | 1: 10 | 10 | MS | S | 11.72 | 13.05 | -1.33 |
| **118** | 1: 10 | 13 | MS | S | 13.38 | 14.22 | -0.84 |

| | | | | | | | |
|---|---|---|---|---|---|---|---|
| **119** | 1: 10 | 15 | MS | S | 14.46 | 14.89 | -0.43 |
| **120** | 1: 10 | 17 | MS | S | 15.16 | 15.49 | -0.32 |
| **121** | 1: 10 | 20 | MS | S | 15.72 | 16.24 | -0.52 |
| **122** | 1: 10 | 22 | MS | S | 15.99 | 16.64 | -0.65 |
| **123** | 1: 10 | 24 | MS | S | 16.28 | 16.97 | -0.70 |
| **124** | 1: 10 | 27 | MS | S | 16.92 | 17.34 | -0.42 |
| **125** | 1: 10 | 29 | MS | S | 17.14 | 17.51 | -0.37 |
| **126** | 1: 10 | 31 | MS | S | 17.14 | 17.61 | -0.46 |
| **127** | 1: 10 | 34 | MS | S | 17.14 | 17.64 | -0.49 |
| **128** | 1: 10 | 35 | MS | S | 17.14 | 17.62 | -0.48 |
| **129** | 1: 10 | 37 | MS | S | 17.14 | 17.53 | -0.39 |
| **130** | 1: 10 | 41 | MS | S | 17.14 | 17.20 | -0.06 |
| **131** | 1: 10 | 43 | MS | S | 17.14 | 16.96 | 0.18 |
| **132** | 1: 10 | 44 | MS | S | 17.14 | 16.82 | 0.32 |
| **133** | 1: 10 | 45 | MS | S | 17.14 | 16.67 | 0.47 |
| **134** | 1: 10 | 8 | OS | N S | 4.09 | 4.67 | -0.58 |
| **135** | 1: 10 | 9 | OS | N S | 4.15 | 5.46 | -1.31 |
| **136** | 1: 10 | 10 | OS | N S | 4.24 | 6.23 | -1.99 |
| **137** | 1: 10 | 13 | OS | N S | 4.80 | 8.41 | -3.61 |
| **138** | 1: 10 | 15 | OS | N S | 5.68 | 9.76 | -4.08 |
| **139** | 1: 10 | 17 | OS | N S | 6.64 | 11.03 | -4.39 |
| **140** | 1: 10 | 20 | OS | N S | 8.96 | 12.78 | -3.82 |
| **141** | 1: 10 | 22 | OS | N S | 10.52 | 13.85 | -3.33 |
| **142** | 1: 10 | 24 | OS | N S | 11.81 | 14.85 | -3.04 |
| **143** | 1: 10 | 27 | OS | N S | 13.86 | 16.21 | -2.34 |
| **144** | 1: 10 | 29 | OS | N S | 15.15 | 17.03 | -1.88 |
| **145** | 1: 10 | 31 | OS | N S | 16.31 | 17.78 | -1.47 |
| **146** | 1: 10 | 34 | OS | N S | 17.43 | 18.79 | -1.35 |
| **147** | 1: 10 | 35 | OS | N S | 17.69 | 19.09 | -1.40 |
| **148** | 1: 10 | 37 | OS | N S | 18.12 | 19.66 | -1.54 |
| **149** | 1: 10 | 41 | OS | N S | 18.55 | 20.62 | -2.07 |
| **150** | 1: 10 | 43 | OS | N S | 18.82 | 21.01 | -2.19 |
| **151** | 1: 10 | 44 | OS | N S | 18.82 | 21.19 | -2.37 |
| **152** | 1: 10 | 45 | OS | N S | 18.82 | 21.36 | -2.54 |
| **153** | 1: 5 | 8 | MS | N S | 2.97 | 1.55 | 1.42 |
| **154** | 1: 5 | 9 | MS | N S | 2.97 | 1.96 | 1.00 |
| **155** | 1: 5 | 10 | MS | N S | 2.97 | 2.36 | 0.61 |
| **156** | 1: 5 | 13 | MS | N S | 3.30 | 3.44 | -0.14 |
| **157** | 1: 5 | 15 | MS | N S | 3.62 | 4.08 | -0.45 |
| **158** | 1: 5 | 17 | MS | N S | 3.62 | 4.64 | -1.02 |
| **159** | 1: 5 | 20 | MS | N S | 3.62 | 5.37 | -1.75 |

| | | | | | | | |
|---|---|---|---|---|---|---|---|
| **160** | 1: 5 | 22 | MS | N S | 3.62 | 5.78 | -2.16 |
| **161** | 1: 5 | 24 | MS | N S | 3.62 | 6.13 | -2.51 |
| **162** | 1: 5 | 27 | MS | N S | 3.62 | 6.55 | -2.93 |
| **163** | 1: 5 | 29 | MS | N S | 3.62 | 6.76 | -3.14 |
| **164** | 1: 5 | 31 | MS | N S | 3.62 | 6.92 | -3.30 |
| **165** | 1: 5 | 34 | MS | N S | 3.62 | 7.06 | -3.44 |
| **166** | 1: 5 | 35 | MS | N S | 3.62 | 7.09 | -3.47 |
| **167** | 1: 5 | 37 | MS | N S | 3.62 | 7.10 | -3.48 |
| **168** | 1: 5 | 41 | MS | N S | 3.62 | 7.00 | -3.38 |
| **169** | 1: 5 | 43 | MS | N S | 3.62 | 6.89 | -3.27 |
| **170** | 1: 5 | 44 | MS | N S | 3.62 | 6.82 | -3.20 |
| **171** | 1: 5 | 45 | MS | N S | 3.62 | 6.75 | -3.13 |
| **172** | 1: 5 | 8 | MS | S | 9.47 | 10.66 | -1.19 |
| **173** | 1: 5 | 9 | MS | S | 10.30 | 11.09 | -0.80 |
| **174** | 1: 5 | 10 | MS | S | 10.87 | 11.50 | -0.64 |
| **175** | 1: 5 | 13 | MS | S | 12.22 | 12.61 | -0.39 |
| **176** | 1: 5 | 15 | MS | S | 13.10 | 13.25 | -0.15 |
| **177** | 1: 5 | 17 | MS | S | 13.89 | 13.82 | 0.07 |
| **178** | 1: 5 | 20 | MS | S | 14.68 | 14.53 | 0.15 |
| **179** | 1: 5 | 22 | MS | S | 15.01 | 14.91 | 0.09 |
| **180** | 1: 5 | 24 | MS | S | 15.33 | 15.23 | 0.10 |
| **181** | 1: 5 | 27 | MS | S | 15.75 | 15.59 | 0.16 |
| **182** | 1: 5 | 29 | MS | S | 15.99 | 15.74 | 0.24 |
| **183** | 1: 5 | 31 | MS | S | 16.14 | 15.84 | 0.29 |
| **184** | 1: 5 | 34 | MS | S | 16.39 | 15.88 | 0.51 |
| **185** | 1: 5 | 35 | MS | S | 16.39 | 15.86 | 0.53 |
| **186** | 1: 5 | 37 | MS | S | 16.59 | 15.79 | 0.80 |
| **187** | 1: 5 | 41 | MS | S | 16.75 | 15.50 | 1.26 |
| **188** | 1: 5 | 43 | MS | S | 16.96 | 15.28 | 1.68 |
| **189** | 1: 5 | 44 | MS | S | 16.96 | 15.15 | 1.81 |
| **190** | 1: 5 | 45 | MS | S | 16.96 | 15.01 | 1.95 |
| **191** | 1: 5 | 8 | OS | N S | 3.63 | 1.47 | 2.16 |
| **192** | 1: 5 | 9 | OS | N S | 3.78 | 2.17 | 1.61 |
| **193** | 1: 5 | 10 | OS | N S | 3.99 | 2.85 | 1.14 |
| **194** | 1: 5 | 13 | OS | N S | 7.14 | 4.75 | 2.39 |
| **195** | 1: 5 | 15 | OS | N S | 8.99 | 5.92 | 3.07 |
| **196** | 1: 5 | 17 | OS | N S | 10.00 | 7.01 | 2.99 |
| **197** | 1: 5 | 20 | OS | N S | 10.88 | 8.51 | 2.38 |
| **198** | 1: 5 | 22 | OS | N S | 11.18 | 9.41 | 1.77 |
| **199** | 1: 5 | 24 | OS | N S | 11.50 | 10.25 | 1.26 |
| **200** | 1: 5 | 27 | OS | N S | 12.37 | 11.37 | 1.00 |

| | | | | | | | |
|---|---|---|---|---|---|---|---|
| **201** | 1: 5 | 29 | OS | N S | 13.09 | 12.04 | 1.05 |
| **202** | 1: 5 | 31 | OS | N S | 13.76 | 12.64 | 1.11 |
| **203** | 1: 5 | 34 | OS | N S | 14.88 | 13.44 | 1.44 |
| **204** | 1: 5 | 35 | OS | N S | 15.20 | 13.68 | 1.53 |
| **205** | 1: 5 | 37 | OS | N S | 15.53 | 14.10 | 1.42 |
| **206** | 1: 5 | 41 | OS | N S | 15.87 | 14.80 | 1.06 |
| **207** | 1: 5 | 43 | OS | N S | 16.15 | 15.08 | 1.07 |
| **208** | 1: 5 | 44 | OS | N S | 16.25 | 15.19 | 1.05 |
| **209** | 1: 5 | 45 | OS | N S | 16.30 | 15.30 | 1.00 |
| **210** | 1: 2.5 | 8 | MS | N S | 3.08 | 2.71 | 0.37 |
| **211** | 1: 2.5 | 9 | MS | N S | 3.15 | 3.32 | -0.17 |
| **212** | 1: 2.5 | 10 | MS | N S | 3.23 | 3.92 | -0.70 |
| **213** | 1: 2.5 | 13 | MS | N S | 3.98 | 5.63 | -1.66 |
| **214** | 1: 2.5 | 15 | MS | N S | 5.70 | 6.69 | -0.99 |
| **215** | 1: 2.5 | 17 | MS | N S | 7.53 | 7.70 | -0.16 |
| **216** | 1: 2.5 | 20 | MS | N S | 9.81 | 9.10 | 0.72 |
| **217** | 1: 2.5 | 22 | MS | N S | 10.87 | 9.96 | 0.91 |
| **218** | 1: 2.5 | 24 | MS | N S | 11.61 | 10.77 | 0.84 |
| **219** | 1: 2.5 | 27 | MS | N S | 12.47 | 11.90 | 0.57 |
| **220** | 1: 2.5 | 29 | MS | N S | 12.98 | 12.59 | 0.38 |
| **221** | 1: 2.5 | 31 | MS | N S | 13.44 | 13.24 | 0.20 |
| **222** | 1: 2.5 | 34 | MS | N S | 14.59 | 14.14 | 0.45 |
| **223** | 1: 2.5 | 35 | MS | N S | 15.21 | 14.42 | 0.79 |
| **224** | 1: 2.5 | 37 | MS | N S | 16.03 | 14.95 | 1.08 |
| **225** | 1: 2.5 | 41 | MS | N S | 16.86 | 15.91 | 0.96 |
| **226** | 1: 2.5 | 43 | MS | N S | 17.20 | 16.34 | 0.86 |
| **227** | 1: 2.5 | 44 | MS | N S | 17.30 | 16.54 | 0.76 |
| **228** | 1: 2.5 | 45 | MS | N S | 17.36 | 16.74 | 0.62 |
| **229** | 1: 2.5 | 8 | MS | S | 5.25 | 5.55 | -0.30 |
| **230** | 1: 2.5 | 9 | MS | S | 6.08 | 6.09 | -0.01 |
| **231** | 1: 2.5 | 10 | MS | S | 6.98 | 6.61 | 0.37 |
| **232** | 1: 2.5 | 13 | MS | S | 8.85 | 8.05 | 0.79 |
| **233** | 1: 2.5 | 15 | MS | S | 9.81 | 8.93 | 0.88 |
| **234** | 1: 2.5 | 17 | MS | S | 10.66 | 9.74 | 0.92 |
| **235** | 1: 2.5 | 20 | MS | S | 11.46 | 10.83 | 0.63 |
| **236** | 1: 2.5 | 22 | MS | S | 11.99 | 11.48 | 0.51 |
| **237** | 1: 2.5 | 24 | MS | S | 12.42 | 12.07 | 0.34 |
| **238** | 1: 2.5 | 27 | MS | S | 12.93 | 12.85 | 0.08 |
| **239** | 1: 2.5 | 29 | MS | S | 13.23 | 13.30 | -0.07 |
| **240** | 1: 2.5 | 31 | MS | S | 13.42 | 13.70 | -0.28 |
| **241** | 1: 2.5 | 34 | MS | S | 13.73 | 14.20 | -0.48 |

| | | | | | | | |
|---|---|---|---|---|---|---|---|
| **242** | 1: 2.5 | 35 | MS | S | 13.81 | 14.34 | -0.54 |
| **243** | 1: 2.5 | 37 | MS | S | 14.03 | 14.60 | -0.57 |
| **244** | 1: 2.5 | 41 | MS | S | 14.19 | 14.97 | -0.79 |
| **245** | 1: 2.5 | 43 | MS | S | 14.32 | 15.10 | -0.78 |
| **246** | 1: 2.5 | 44 | MS | S | 14.32 | 15.15 | -0.83 |
| **247** | 1: 2.5 | 45 | MS | S | 14.32 | 15.19 | -0.87 |
| **248** | 1: 2.5 | 8 | OS | N S | 4.01 | 3.10 | 0.92 |
| **249** | 1: 2.5 | 9 | OS | N S | 4.09 | 3.76 | 0.34 |
| **250** | 1: 2.5 | 10 | OS | N S | 4.17 | 4.39 | -0.22 |
| **251** | 1: 2.5 | 13 | OS | N S | 5.00 | 6.20 | -1.20 |
| **252** | 1: 2.5 | 15 | OS | N S | 5.96 | 7.31 | -1.34 |
| **253** | 1: 2.5 | 17 | OS | N S | 6.62 | 8.35 | -1.73 |
| **254** | 1: 2.5 | 20 | OS | N S | 8.60 | 9.79 | -1.19 |
| **255** | 1: 2.5 | 22 | OS | N S | 9.75 | 10.66 | -0.92 |
| **256** | 1: 2.5 | 24 | OS | N S | 11.12 | 11.48 | -0.35 |
| **257** | 1: 2.5 | 27 | OS | N S | 12.59 | 12.58 | 0.00 |
| **258** | 1: 2.5 | 29 | OS | N S | 13.47 | 13.25 | 0.21 |
| **259** | 1: 2.5 | 31 | OS | N S | 14.20 | 13.86 | 0.34 |
| **260** | 1: 2.5 | 34 | OS | N S | 15.14 | 14.68 | 0.45 |
| **261** | 1: 2.5 | 35 | OS | N S | 15.48 | 14.93 | 0.54 |
| **262** | 1: 2.5 | 37 | OS | N S | 15.82 | 15.39 | 0.42 |
| **263** | 1: 2.5 | 41 | OS | N S | 16.16 | 16.18 | -0.02 |
| **264** | 1: 2.5 | 43 | OS | N S | 16.28 | 16.50 | -0.22 |
| **265** | 1: 2.5 | 44 | OS | N S | 16.31 | 16.65 | -0.35 |
| **266** | 1: 2.5 | 45 | OS | N S | 16.32 | 16.79 | -0.47 |

**Abbreviations:** Exp: experiment; S: supplemented; NS: Not supplemented.

*Actual and predicted values are square roots of methane concentration (ml/g $VS^{1/2}$)
